# Combined central retinal artery occlusion and vein occlusion with exudative retinal detachment following COVID‐19 vaccination

**DOI:** 10.1002/kjm2.12591

**Published:** 2022-09-13

**Authors:** Yen‐Chih Chen

**Affiliations:** ^1^ Department of Ophthalmology Yunlin Christian Hospital Xiluo Taiwan; ^2^ Department of Optometry Central Taiwan University of Science and Technology Taichung Taiwan

Due to the coronavirus disease 2019 (COVID‐19) pandemic, various vaccines are being administered worldwide to combat the menace. However, increasing literature has reported postvaccine‐related ocular thromboembolic events, including retinal artery/vein occlusion, ischemic optic neuropathy, and combined presentations.^1^, ^2^ Here, we report a rare combination of central retinal artery occlusion (CRAO), central retinal vein occlusion (CRVO), and exudative retinal detachment following the COVID‐19 vaccine.

A 72‐year‐old male presented to our hospital due to sudden severe vision loss in his right eye 2 days ago. Investigations revealed that he was a nonsmoker and his previous medical history was unremarkable, only having received a second dose of the COVID‐19 vaccine (BNT162b2; Pfizer‐BioNTech, New York, NY, and Mainz, Germany) 10 days ago. He has self COVID‐19 rapid antigen test and the result was negative.

Upon his initial visit, his right eye visual acuity was hand motion, and 20/25 of his left eye. The intraocular pressure was 11 mm Hg in the right eye and 14 mm Hg in the left eye, and anterior segment examinations were normal in both eyes. Right eye fundus examination showed extensive flame‐shaped hemorrhages, attenuated retinal arteries, and whitening of the macula (Figure 1A). Optical coherence tomography (OCT) indicated hyper‐reflectivity of inner retinal layers, loss of the retina structure, and massive subretinal fluid at macular area (Figure 1B). Based on these findings, we diagnosed the patient with combined CRAO and CRVO, exudative retinal detachment at posterior pole. Immediate fluorescein angiography (FA) showed delayed perfusion filling with wide non‐perfused areas. Leaking vessels were found at the periphery as well as disc area (Figure 1C). Laboratory tests including complete blood count, erythrocyte sedimentation rate, C‐reactive protein, hemoglobin A1c, lipid profile, prothrombin time, anti‐cardiolipin antibody, lupus anticoagulant, protein C/protein S activity, treponema, and quantiferon test results were all normal, except for elevated D‐dimer value(547 ng/ml). He had normal blood pressure and heart rate. Carotid sonography and echocardiography were unremarkable. He received an intravitreal injection of aflibercept (2 mg/0.05 ml, EYLEA®) and intravenous methylprednisolone (1 g/day) for 3 days. After treatment, the subretinal fluid was reabsorbed, then oral prednisolone (1 g/kg/day) and aspirin were administered and tapered according to the clinical condition. Panretinal photocoagulation was also applied. One month later, the right eye vision improved to 20/400. OCT scans confirmed the attachment of the retina, but with loss of the retina structure (Figure 1D).

**FIGURE 1:**
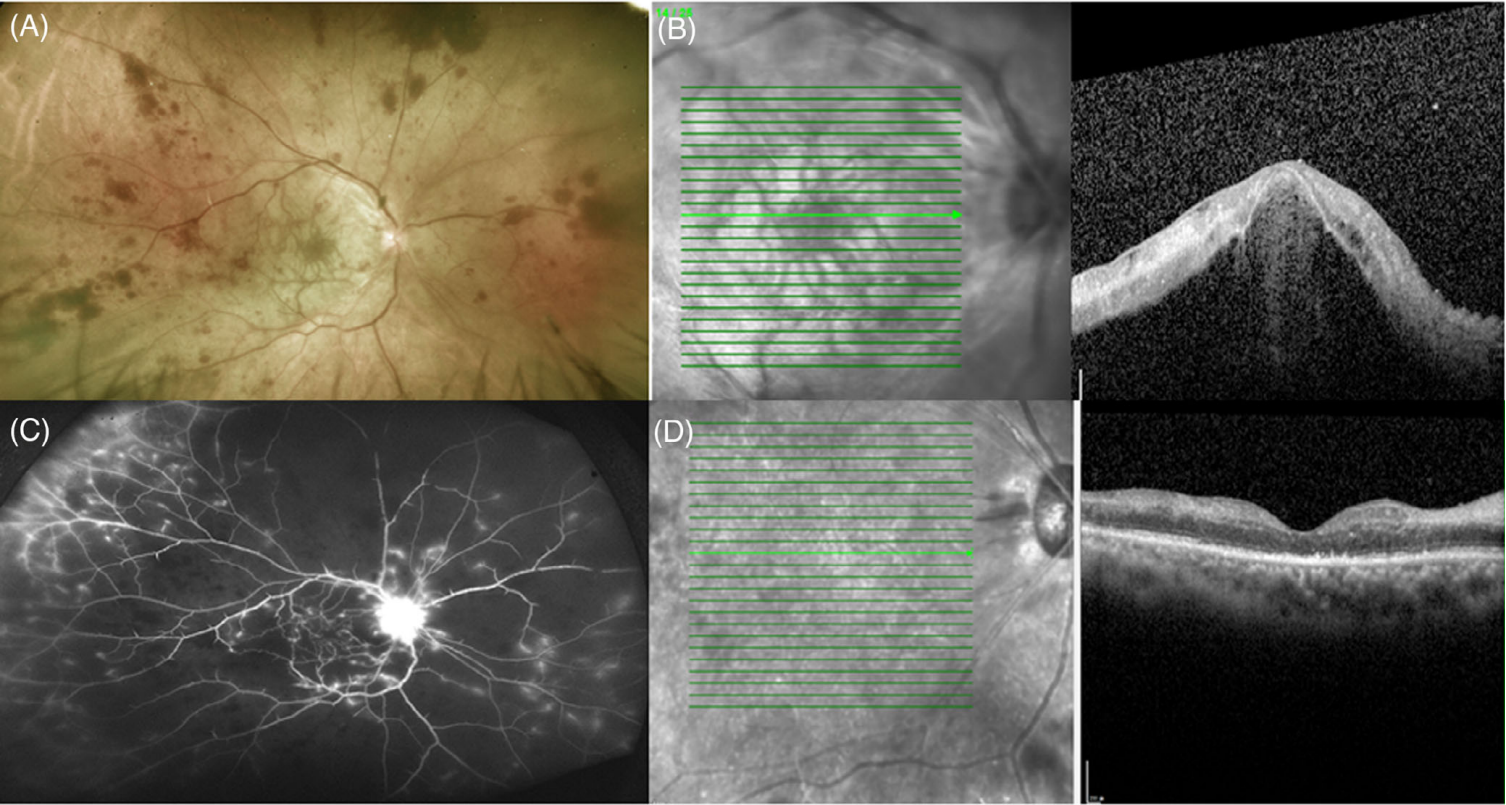
Combined central retinal artery/vein occlusion and exudative retinal detachment of right eye after COVID‐19 vaccination. (A) Fundoscopic examination showed extensive retina hemorrhages, attenuated artery, and whitening of macular area. (B) Optical coherence tomography (OCT) demonstrated retinal detachment and hyper‐reflective change of inner retina. (C) Late‐phase fluorescein angiography showed wide non‐perfused area and peripheral vessel as well as disc fluorescein leakage. (D) 1 month after the treatment, OCT revealed subsided subretinal fluid, but a disorganized and atrophic retina structure

Combined CRAO and CRVO is a rare but devastating ocular complication, leading to severe visual loss and poor visual prognosis. Vessel occlusions are usually associated with several risk factors, including age, hypertension, dyslipidemia, diabetes mellitus, glaucoma, vasculitis, or systemic hypercoagulable state related to rheumatology diseases or ocular infection. Here we report a case of combined CRAO with CRVO, not caused by any risk factor but concomitantly accompanied by exudative retinal detachment in a patient following COVID‐19 vaccination. While we could not establish a definitive cause and effect between the vaccine and the observed symptoms, considering the short period between both events and, including the fact that all possible risk factors were negative, a correlation between the COVID‐19 vaccine and the observed vessel occlusion may be suggested.

More cases have been reported with systemic thrombotic complications associated with COVID vaccination,^3^ and both venous and arterial thrombotic events have also been observed following various COVID‐19 vaccines.^4^ These complications are believed to result from inflammation‐induced thrombosis. From our case, leaking vessels were observed in FA imaging, indicating the presence of retinal vasculitis. And the subretinal fluid, high in the understudied patient, reflected the inflammation severity. Systemic corticosteroids and intravitreous injection of aflibercept are believed to reduce the inflammation in retinal vein occlusion. After treatment, the exudative retinal detachment improved, followed by visual acuity improvement. However, the visual prognosis was guarded because of the sequela of the retina vessel occlusion.^5^

Vaccination has been established as the gold standard for preventing the high mortality and morbidity rates associated with COVID‐19. However, we report a severe ocular adverse event proposed to be caused by vaccination. Further observations should validate this potential association.

## CONFLICT OF INTEREST

The author declares that there is no conflict of interest.
